# Rationale for Considering Oral Idasanutlin as a Therapeutic Option for COVID-19 Patients

**DOI:** 10.3389/fphar.2020.01156

**Published:** 2020-07-29

**Authors:** Giorgio Zauli, Veronica Tisato, Paola Secchiero

**Affiliations:** Department of Morphology, Surgery and Experimental Medicine and LTTA Centre, University of Ferrara, Ferrara, Italy

**Keywords:** SARS-CoV-2, COVID-19, p53, MDM2, Idasanutlin, antiviral activity, new therapeutic approach

## Introduction

The spread of the novel severe acute respiratory syndrome coronavirus 2 (SARS-CoV-2), firstly detected in Wuhan (China) at the end of 2019, has rapidly reached pandemic status. At the present time, under the “pressure” of the infection the need of effective and innovative treatments is becoming crucial. In particular, new insights into potential innovative therapeutic interventions, including the repositioning of pharmaceutical compounds already available that may be useful to control and contrast coronavirus disease 2019 (COVID-19) caused by SARS-CoV-2 are needed, also in the view of potential second waves of infection. With this aim, we here describe the rationale for the use of Idasanutlin, an orally available, potent and selective small-molecule antagonist of MDM2 acting as non-genotoxic p53 activator to treat COVID-19 patients and support its clinical evaluation.

## Discussion

Under physiological conditions, the levels of the “guardian of genome” p53 are kept low in cells mainly through the binding of its principal inhibitor murine double minute 2 (MDM2), which acts on p53 through a well-characterized negative feedback. Viruses are known to induce a plethora of stress signals leading to post-translational modifications of p53 and of its main negative regulator MDM2, interfering with the normal p53/MDM2 regulatory loop (Sato and Tsurumi, 2013). If the regulatory role of MDM2 is lost, the intracellular p53 levels increase leading to well-characterized activation pathways with a distinctive gene expression signature. In particular, this results into cell cycle arrest, which if long lasting leads to apoptosis, and to a variety of cellular responses (Secchiero et al., 2006).

Coronaviruses are viruses with envelope and a single stranded, 5′-capped, positive strand RNA molecule (26–32 kb) with at least six open reading frames (Perlman and Netland, 2009). After cell infection, the genomic RNA is translated into two polyproteins, pp1a and pp1ab that are further processed by cysteine proteases of viral origin, such as papain-like proteases (PLPs) (Perlman and Netland, 2009). Interestingly, in addition to processing viral polyproteins, it has been reported that PLP2 of HCoV-NL63 (i.e. human coronavirus NL63), can act as MDM2 stabilizer (via deubiquitination) leading in turn to p53 proteasomal degradation (Yuan et al., 2015). Of note, Middle East Respiratory Syndrome (MERS)-CoV (MERS-CoV) PLpro cause endogenous p53 degradation by similar mechanisms (Ma-Lauer et al., 2016). The PLP encoded by SARS-CoV-2 nsp3 has 86% amino acid homology with SARS-CoV PLP sequence (Dong et al., 2020). Thus, it has been inferred that also SARS-CoV-2 PLP uses its protein hydrolase activity and de-ubiquitinase activity to evade the host’s antiviral immune response and inhibit the expression of interferon (Dong et al., 2020). Therefore, SARS-CoV, MERS-CoV, and SARS-CoV-2 PLP are all able to mediate p53 degradation in a process that lead to decreased cell apoptosis favoring viral growth in infected cells as well as to the loss of the antiviral activity of p53.

In this regard, p53 is a pleiotropic molecule deeply analyzed for its several functions and involvement in different pathways, including those related to antiviral innate immune responses carried out particularly by inducing apoptosis of infected cells and mediating type I interferon (IFN) production/signaling (Munoz-Fontela et al., 2008). In the context of antiviral immunity, p53 plays therefore a critical role and perhaps this is why it represents a frequent viral target. In this line, it is of interest that viruses with a strong airway epithelial cells tropism such as the respiratory syncytial virus (RSV) activate MDM2 *via* PI3K/Akt within the few hours after infection, leading to a fast decrease in p53 levels that results in airways epithelial cell survival (Groskreutz et al., 2007). Interestingly, the prolonged survival of virus-target cells did not increase viral replication but rather exacerbated inflammation as demonstrated by higher levels of key inflammatory mediators such as interleukin (IL)-6 (Groskreutz et al., 2007), i.e. key cytokine of the “cytokine storm” characterizing COVID-19 patients. The onset of unrestrained release of inflammatory cytokines/chemokines characterizes severest COVID-19 patients as reported by Huang and colleagues when comparing intensive care unit (ICU) patients to non-ICU patients (Huang et al., 2020), and afterward confirmed as key feature of the disease in several studies. The so called “cytokine storm” in COVID-19 is therefore strongly associated with disease severity and responsible for the wide range of symptoms going from asymptomatic to fatal symptomatic cases, in which biological sex, age and inherited predispositions are also involved (Gemmati et al., 2020).

Of interest, the protective role of p53 in counteracting acute respiratory distress syndrome has been recently demonstrated *in vivo* in P53 knockout mice that triggered more severe inflammatory responses when challenged with LPS compared to wild type littermates (Uddin et al., 2020). In this line, both RSV-mediated cell survival and inflammatory burden resulted antagonized by Nutlin-3 treatment in *in vitro* cell models (Groskreutz et al., 2007). The potential of MDM2 antagonists in attenuating the association between cell-senescence and inflammatory processes has been recently investigated at preclinical level (Wiley et al., 2018). Small-molecules inhibitors of MDM2 such as Nutlin-3 and MI-63 by promoting p53 survival can be useful to reduce the so-called “senescence-associated secretory phenotype” and lowering in particular IL-6 secretion and the overall pro-inflammatory burden (Wiley et al., 2018).

Indirect suggestions that SARS-CoV-2 may affect the MDM2/p53 regulatory loop comes from the evidence that similarly to SARS-CoV and MERS-CoV (Chen and Subbarao, 2007; Yuan et al., 2015), the new coronavirus induces low type I IFNs levels, most likely contributing to slow-down the immune response in COVID-19 patients (Li et al., 2020).

Idasanutlin is a second-generation potent and selective small-molecule MDM2 antagonist with a pyrrolidine structure (Ding et al., 2013). Idasanutlin shows an identical cellular mechanism to other Nutlin family molecules, which our group of investigators has intensively studied over more than a decade both in *in vitro* and *in vivo* models as non-genotoxic activators of p53 (Tisato et al., 2017). Compared to first-generation Nutlin, second-generation Idasanutlin showed enhanced potency, selectivity, and bioavailability (Ding et al., 2013). In a multicenter clinical study of phase I/Ib, administration of Idasanutlin at doses 400–1600 mg/d for 5 d to AML patients showed acceptable safety, supporting its clinical evaluation as monotherapy and in combination with anti-leukemic drugs (Montesinos et al., 2020). In another recent study on policytemia vera, patients were treated with Idasanutlin (doses: 100 and 150 mg/d respectively) following a schedule of treatments of 5 consecutive days of a 28-d cycle (Mascarenhas et al., 2019), and Idasanutlin was well tolerated. Overall, the study did not show dose-limiting toxicity, although low-grade gastrointestinal toxicity was commonly detected (Mascarenhas et al., 2019). Of note, a recent review confirmed that Idasanutlin is well tolerated (Khurana and Shafer, 2019). With regard to common negative side effects due to Idasanutlin treatment, the reported studies were restricted to diarrhea, nausea/vomiting and in some cases myelosuppression causing febrile neutropenia and thrombocytopenia (Siu et al., 2014), thought considered to be the effect of the drug on the normal cells (Tisato et al., 2017).

**Figure 1:**
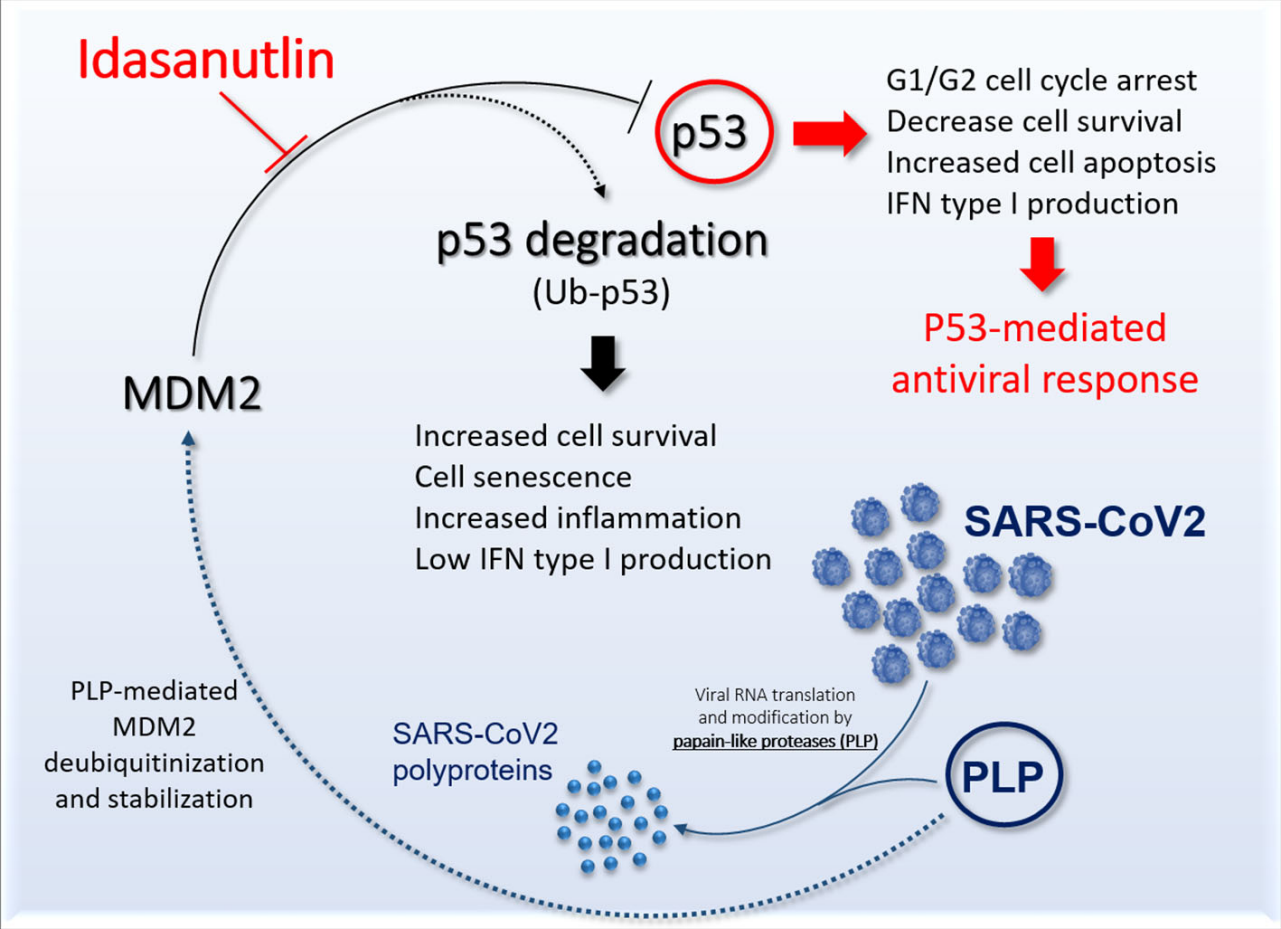
Schematic representation the potential role of Idasanutlin to restore functional p53 antiviral activity. The picture shows the link between SARS-CoV-2 PLP and murine double minute 2 (MDM2) leading to inhibition of p53 antiviral activity and the potential role of Idasanutlin in disrupting this regulatory loop and reestablishing functional p53 activity.

On these bases, we believe that Idasanutlin represents an important candidate molecule to counteract SARS-CoV-2 pneumonia (**Figure 1**) and it should be tested in clinical trials in symptomatic COVID-19 patients.

## Author Contributions

Conceptualization: GZ. Writing—original draft preparation: GZ, VT, and PS. Writing—review and editing: GZ, VT, and PS.

## Conflict of Interest

The authors declare that the research was conducted in the absence of any commercial or financial relationships that could be construed as a potential conflict of interest.
